# Screening for Human Immunodeficiency Virus in Inner City Females With Abnormal Cervical Cytology

**DOI:** 10.1155/S1064744996000543

**Published:** 1996

**Authors:** T. Scott Jennings, Peter Dottino, Rhoda Sperling, Ann Marie Beddoe

**Affiliations:** 1 Department of Obstetrics, Gynecology, and Reproductive Sciences Mount Sinai Hospital New York New York USA; 2 Division of Gynecologic Oncology Department of Obstetrics and Gynecology Medical University of South Carolina 171 Ashley Avenue Charleston SC 29425 USA

## Abstract

*Objective:* This report evaluates the acceptance, results, and predictors of human immunodeficiency virus (HIV) infection in inner city women referred to a colposcopy clinic for abnormal cervical cytology.

*Methods:* HIV testing results of 1,908 inner city women referred for abnormal cervical cytology were analyzed retrospectively with respect to acceptance, race, ethnicity, Pap smear results, sexually transmitted diseases (STDs), HIV exposures, and final histologic findings.

*Results:* HIV testing was accepted by 50.4% of patients. Women who agreed to screening were significantly more likely to admit exposure to HIV or to be Hispanic, foreign-born, or have a history of multiple STDs. Of those screened, 3.3% were found to be HIV seropositive. Although higher grades of referral Pap smears were noted in the women found to be HIV seropositive, final histologic findings were not different. The only predictors of unknown HIV seropositivity were admitted HIV exposure and external condyloma.

*Conclusions:* Fifty percent of inner city women of unknown HIV status referred for abnormal cervical cytology will accept HIV serotesting and 3.3% are found to be positive. Most HIV-seropositive women can be detected based on either a history of exposure to HIV or the presence of external condyloma.


Infectious Diseases in Obstetrics and Gynecology 4:281–286 (1996)
© 1997 Wiley-Liss, Inc.

### Screening for Human Immunodeficiency Virus in Inner City Females With Abnormal Cervical Cytology

T. Scott Jennings,* Peter Dottino, Rhoda Sperling, and Ann Marie Beddoe

*Department of Obstetrics, Gynecology, and Reproductive Sciences, Mount Sinai Hospital, New York, New York*

ABSTRACT

*Objective:* This report evaluates the acceptance, results, and predictors of human immunodeficiency virus (HIV) infection in inner city women referred to a colposcopy clinic for abnormal cervical cytology.

*Methods:* HIV testing results of 1,908 inner city women referred for abnormal cervical cytology were analyzed retrospectively with respect to acceptance, race, ethnicity, Pap smear results, sexually transmitted diseases (STDs), HIV exposures, and final histologic findings.

*Results:* HIV testing was accepted by 50.4% of patients. Women who agreed to screening were significantly more likely to admit exposure to HIV or to be Hispanic, foreign-born, or have a history of multiple STDs. Of those screened, 3.3% were found to be HIV seropositive. Although higher grades of referral Pap smears were noted in the women found to be HIV seropositive, final histologic findings were not different. The only predictors of unknown HIV seropositivity were admitted HIV exposure and external condyloma.

*Conclusions:* Fifty percent of inner city women of unknown HIV status referred for abnormal cervical cytology will accept HIV serotesting and 3.3% are found to be positive. Most HIV-seropositive women can be detected based on either a history of exposure to HIV or the presence of external condyloma. Infect. Dis. Obstet. Gynecol. 4:281–286, 1996. © 1997 Wiley-Liss, Inc.

KEY WORDS

HIV testing; abnormal cervical cytology; CIN; external condyloma

An association between human papillomavirus (HPV)-related diseases of the cervix and human immunodeficiency virus (HIV) infection has been recognized.[1] HIV-infected persons have been reported in several series to have increased rates of HPV expression, increased rates of HPV-associated intraepithelial lesions, and increased failure rates following standard treatment for intraepithelial diseases.[2–4] Reports of more aggressive clinical courses in patients with invasive cervical cancer[5] have led to inclusion of cervical cancer as an acquired immunodeficiency syndrome (AIDS)-defining illness.[6] Additionally, HIV screening has been recommended in patients with HPV-associated intraepithelial and invasive neoplasms.[7] However, the extent to which HIV-infected individuals have contributed to an excess burden of HPV-related diseases is unclear.[8]

In 1988, anonymous serum testing in New York found 10% of women referred to an inner city colposcopy clinic to be HIV seropositive.[9] A preliminary report from our colposcopy clinic, located in a teaching hospital serving primarily the East Harlem community of New York, found 13 of 261

*Correspondence to: Dr. T. Scott Jennings, Division of Gynecologic Oncology, Department of Obstetrics and Gynecology, Medical University of South Carolina, 171 Ashley Avenue, Charleston, SC 29425.

Received 19 April 1996

Accepted 11 November 1996

Clinical Study women of unknown HIV serostatus referred for abnormal cervical cytology to be HIV seropositive.[10] This report expands upon our preliminary report, evaluates factors that predict acceptance of HIV screening, and compares HPV-related disease in HIV-seropositive and HIV-seronegative women.

### SUBJECTS AND METHODS

From January 1, 1990, to December 1, 1994, all women of unknown HIV serostatus referred to the Mount Sinai Hospital for outpatient evaluation of abnormal Pap smears were offered individual counseling and screening for HIV. In compliance with New York State legislation, all HIV counseling and screening services were strictly confidential. Pre- and post-test counseling were conducted by specifically trained personnel immediately following colposcopy. If the patient agreed to HIV screening after counseling, enzyme-linked immunosorbent assay (ELISA) followed by Western blot confirmation of positive results was performed. Separate logbooks compiled computer-coded results that were not linked to patient names to ensure confidentiality.

All patients were queried regarding history of sexually transmitted disease (STD), history of biopsy-proven cervical intraepithelial neoplasia (CIN), and specific exposure to HIV. Unless previously screened at the time of referral for abnormal cervical cytology, patients were screened for syphilis, *Neisseria gonorrhoeae*, and *Chlamydia trachomatis* by cultures or serology. At the time of initial evaluation, the lower genital tract was carefully inspected for gross evidence of external condyloma. A 3% acetic acid solution was used to cleanse the cervix, after which the upper vagina and cervix were visualized colposcopically with a 10× to 25× lens. All colposcopic evaluations were performed by residents in gynecology directly supervised by specialists in gynecologic oncology. Biopsies and endocervical curettage were performed in patients in whom colposcopic evidence of CIN was seen. Colposcopic evaluation and biopsies were performed without regard to the results of HIV counseling and screening. During subsequent visits, colposcopy and treatment were generally conducted with knowledge of HIV test results.

Results were retrospectively compiled in a personal computer data base using both clinic records and record of HIV serotesting. Comparative analyses, including chi-square analysis and analysis of variance (ANOVA), were performed with StatView 4.01 (Abacus Concepts, Berkeley, CA) with a significance level of $P = 0.05$. For estimation of the predictive value for HIV seropositivity of selected characteristics, odds ratios (ORs) with 95% confidence intervals (CIs) were calculated.[13]

### RESULTS

### Evaluation of All Patients

During the time of this study, 2,101 patients were referred to the Mount Sinai Hospital Colposcopy Clinic for colposcopic examination. Of these, 125 were known to be HIV seropositive at the time of referral and were excluded. An additional 68 records had insufficient clinical material for evaluation, leaving 1,908 patients for evaluation. Approximately 67% of the patients were referred from hospital-based clinics, 25% from health department clinics, and 8% from private physicians. The majority of women were referred for low grade squamous intraepithelial lesions (LGSIL) (50%); lesser numbers were referred for high grade squamous intraepithelial lesions (HGSIL) (14%) or cancer (0.7%) on Pap smear.

The population was predominantly young minority women, with a mean age of 29.3 years; only 8.6% were postmenopausal. Fifty-five percent were Hispanic, 38% were African American, 7% were white, and 0.3% were of other race. The median gravidity was two and median parity was one. Reported current contraceptive practices of the women included barrier methods (28%), oral contraceptives (21%), parenteral hormones (6%), surgical sterilization (8%), and no contraception (38%). Only 1.7% of the population recalled a history of biopsy-confirmed CIN, but 22.5% reported a history of either gonorrhea, *Chlamydia,* or syphilis. Five percent of the women in this clinic population admitted current or past behavior which placed them at risk for HIV acquisition, including injection drug use (IDU) in 1.4% and heterosexual contact with men who were known or strongly suspected to be HIV-infected in 2.9%. A small number of women (0.7%) reported other risks of HIV exposure, including transfusion of blood products between 1978 and 1985, living in an institutional setting with other HIV-seropositive individuals, or occupational exposure.

### Analysis of Patients Based on Acceptance of HIV Screening

Of the 1,908 patients for evaluation, 718 (37.6%) refused both counseling and testing, 229 (12%) agreed to counseling but refused antibody testing, and 961 (50.4%) were counseled and agreed to HIV-1 antibody testing. Women born outside the United States were significantly more likely to agree to HIV screening than United States-born women ($P < 0.0001$). Hispanic women tended to agree to HIV screening more frequently than African Americans ($P = 0.0004$), and even United States-born Hispanics were more likely to accept HIV screening than United States-born African Americans ($P = 0.034$). Both younger and older patients were less likely to agree to HIV screening than patients between the ages of 20 and 59 years. Patients who accepted testing were significantly more likely to admit exposure to HIV infection than were patients who refused testing. The patients who refused HIV testing were more likely to utilize barrier contraceptive methods, but overall use of contraception did not differ based upon the acceptance of HIV testing. No differences were seen among the frequencies of prior CIN, STDs, referral indications, smoking, or drug abuse. STD testing for syphilis, gonorrhea, and *Chlamydia* did not reveal any differences in overall frequency of STDs (5.4% in those who accepted vs. 5.9% in those who refused). Analysis of the patterns of testing and acceptance of HIV testing by year (1990–1994) did not reveal any significant change over the 5 years of this study.

Hispanic women who refused screening reported significantly fewer exposures to HIV ($P = 0.031$) and were less likely to have had a history of STD ($P = 0.008$) than the Hispanic women who agreed to screening. Among non-Hispanic women, no differences were found for the presence or absence of HIV exposure or history of STD based upon the acceptance of HIV testing.

### Results of HIV Screening in Those Who Accepted Testing

Thirty-two patients, or 3.3% of the 961 who agreed to screening, were identified as HIV seropositive. Table 1 demonstrates the comparisons among those found to be HIV seropositive vs. those who were HIV seronegative (one patient remained inconclusive despite repeat testing 4 months later, and was deleted from further analysis). The only differences among these groups of patients were admission of HIV exposure, presence of HGSIL on referring Pap smear, and gross evidence of external condyloma. Among both groups, the most common high risk exposure was heterosexual exposure to HIV-infected men, reported in 79% of those demonstrated to be HIV seropositive.

TABLE 1. Demographic analysis of patients who accepted HIV screening[a]

| | HIV positive (N = 32) | HIV negative (N = 928) | *P* |
|---|---|---|---|
| Median age | 31.4 years | 29.5 years | NS (ANOVA) |
| Premenopausal | 100% | 92.7% | NS |
| Race | | | |
| African American | 25% | 34.2% | NS |
| Hispanic | 71.4% | 59.2% | NS |
| White | 3.6% | 6.4% | NS |
| Referral indications | | | |
| Pap, atypia | 26.1% | 26.5% | NS |
| LGSIL | 44.8% | 53.3% | NS |
| HGSIL | 31.0% | 13.3% | 0.007 |
| Pap, cancer | 0 | 0.7% | NS |
| Pap, HGSIL or cancer | 31.0% | 13.8% | 0.011 |
| External condyloma | 3.4% | 3.8% | NS |
| History of CIN | 0 | 1.5% | NS |
| Any HIV exposure | 43.8% | 4.7% | <0.0001 |
| Any STD history | 33.3% | 20.6% | NS |
| Not United States-born | 18.8% | 21.4% | NS |
| Gross external condyloma | 28.1% | 7.0% | 0.0004 |
| Age at first intercourse | 16.5 years | 16.2 years | NS (ANOVA) |

[a]NS = not significant.

During the 5 years of this study, there were minor fluctuations in the rates of unknown HIV seropositivity found in this population, but in no year did the yield of HIV screening differ from the other years by more than 1%. Patients under the age of 20 years and over the age of 50 years were significantly less likely to be HIV seropositive (less than 0.01%) than those between 20 and 49 years.

Absolute CD4+ lymphocyte counts were available for 17 of the 32 newly diagnosed HIV-seropositive patients. Only 2 of these 17 patients had CD4+ counts less than 200/μl, and no other AIDS-defining illness was noted among the newly diagnosed HIV-seropositive patients within 4 months of diagnosis.

Women found to be HIV seropositive had a

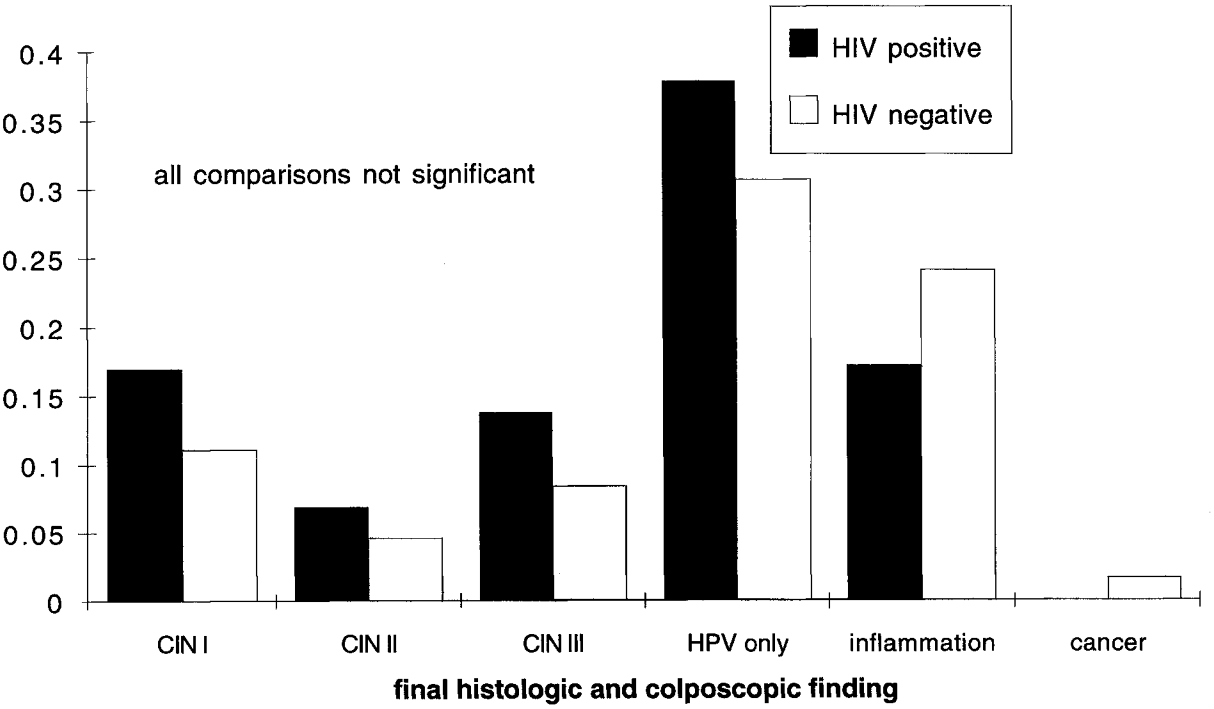

Fig. 1. Comparison of final cervical diagnoses (combined histologic and colposcopic findings) by HIV seropositivity in all patients who accepted HIV testing. Data are expressed in proportions of women in each group.

higher frequency of HGSIL or cancer on referring cervical cytology smears than women demonstrated to be HIV negative (31% vs. 13%; Table 1). The final results of colposcopic evaluation and histologic findings, however, demonstrate no significant differences in the severity of cervical disease between the HIV-seropositive and HIV-seronegative women (Fig. 1). Even among women referred for HGSIL or cancer on Pap smear, colposcopic and histologic findings were similar for the HIV-seropositive and HIV-seronegative women. None of the HIV-seropositive women were found to have cervical cancer, while 12 HIV-seronegative women had cervical cancer (10 squamous cell carcinomas and 2 cervical adenocarcinomas). This difference was not statistically significant.

### Analysis of Predictors of HIV Seropositivity

In an effort to delineate predictors of HIV seropositivity in women with abnormal cervical cytology, ORs with 95% CIs were calculated (Table 2). The strongest risk factor for unknown HIV seropositivity was reported HIV exposure (OR 15.6, 95% CI 7.3–33.4). The presence of external condyloma of the lower genital tract was also significantly associated with the finding of HIV seropositivity (OR 5.8, 95% CI 2.6–13.1). HGSIL on referring cervical cytology had a weak but significant association (OR 2.6, 95% CI 1.2–5.8) with HIV seropositivity when analyzed with all patients. However, when patients who reported exposure to possible HIV infection were omitted in a subset analysis (i.e., analysis of the 903 patients who denied HIV exposure, data not shown), the association of HGSIL on referral smear with HIV seropositivity was no longer significant (95% CI now included one). In this subset

TABLE 2. ORs of predictors of unknown HIV seropositivity in all women referred for abnormal cervical cytology

| | OR | CI |
|---|---|---|
| HIV exposure risk | 15.6 | 7.3–33.4* |
| History of STD | 1.3 | 0.58–3.14 |
| Either history of STD or HIV exposure | 3.3 | 1.6–6.7* |
| Not United States-born | 0.9 | 0.3–2.1 |
| African American race | 0.6 | 0.3–1.5 |
| Hispanic race | 1.73 | 0.8–4.0 |
| White race | 0.54 | 0.1–4.1 |
| Other cervical pathogens[a] | 1.56 | 0.4–6.8 |
| LGSIL on referral cytology | 0.72 | 0.4–1.5 |
| HGSIL or cancer on referral cytology | 2.6 | 1.2–5.8* |
| External condyloma | 5.8 | 2.6–13.1* |

[a]Including *N. gonorrhea*, *Chlamydia*, or syphilis (no prior history).
*Significant at $P < 0.05$.

analysis, the only association with HIV seropositivity in those women who denied exposure to HIV infection was the presence of external condyloma. Thirty-three percent of patients who denied exposure to HIV but were found to be HIV seropositive were noted to have external condyloma at the time of initial colposcopic evaluation. In final analysis, 63% of women in this sample who were found to be HIV seropositive were noted to either admit exposure to HIV or demonstrate gross evidence of external condyloma at the time of referral.

### DISCUSSION

This study demonstrates the feasibility and results of systematic HIV screening in women of unknown HIV serostatus undergoing evaluation for abnormal cervical cytology. In our population from an area of high HIV endemicity, HIV screening was accepted after counseling by approximately 50% of women. This acceptance of HIV screening is similar to the 60% and 67% acceptance rates noted in the previous smaller studies by McCarthy et al.[12] and Spitzer et al.,[13] respectively. Approximately 3% of our screened patients were identified as HIV-infected by voluntary screening. Although this incidence is clearly higher than that of reported seroprevalence studies in female first-time blood donors without reported risk factors,[14] it is also less than most reported prior seroprevalence estimates in urban women referred for abnormal cervical cytology in inner cities.

The seropositive yield of HIV screening in this series was lower than in similar previous studies of urban women. The reports from the boroughs of Brooklyn[15] and Queens[13] in New York City may be different due to socioeconomic risk factors other than abnormal cervical cytology. In the reports from Queens, 8 of 132 inner city patients (6.1%) were newly identified after referral to a colposcopy clinic. In studies from Brooklyn, Fruchter and colleagues[15] extrapolated that 13% of inner city colposcopy clinic patients were HIV seropositive. However, the majority of the HIV-seropositive women in their sample were known to be HIV-infected prior to referral, such that the yield of HIV screening in these 173 patients of unknown status was only 6.4%. In our much larger sample of 961 inner city women of unknown serostatus who agreed to HIV screening, only 3.3% of the patients were found to be HIV seropositive.

In this study, women at higher apparent risk of being HIV seropositive (because of admitted heterosexual HIV exposures) were more likely to participate in HIV testing than those who refused testing. HIV counseling was offered in both English and Spanish, and Hispanic women were found to be more likely to agree to screening. Hispanic women who agreed to screening appeared to be at higher risk of HIV infection based on higher rates of self-reported HIV exposure. Unfortunately, African American women with reported exposures to HIV were equally likely to refuse or accept HIV screening. This suggests HIV screening is poorly accepted by African American women even in the presence of risk factors for HIV infection.

Only one-half of the women who were demonstrated to be HIV seropositive admitted exposure to HIV. By observation of external condyloma as well as exposure to HIV, however, we could have correctly identified approximately two-thirds of our otherwise-unsuspected HIV-seropositive women. The association of external condyloma to HIV seroprevalence in patients referred for abnormal cervical cytology has been noted before.[13,15] In the series from Brooklyn, the OR for condyloma with HIV infection was 3.8 (confidence interval 1.0–14.2), and was even more significant after controlling for age (OR 4.9).[15] In our series, not only was external condyloma strongly associated with the identification of HIV-seropositive patients, but external condyloma was the only independent predictor for the presence of HIV seropositivity in patients who deny or are not aware of exposure to HIV-associated behavior.

Although previous studies have noted associations between HIV infection and high grade CIN/cervical cancer, we saw little evidence of association between biopsy-proven high grade CIN/cancer and previously unsuspected HIV infection. Most previous studies analyzed the cervical disease in patients known to be HIV-infected, whereas our HIV-infected patients were detected by systematic screening. Therefore, those found to be HIV seropositive in this study may be earlier in the natural history of their immunodeficiency than the HIV-infected women described in previous reports. As suggested by Smith et al.,[16] it appears that severity of cervical disease may be more dependent upon the degree of immunodepression than on the presence or absence of HIV infection.

It is possible that the more aggressive and fulminant courses of cervical cancer found in other reports of HIV-infected women may have been due to factors other than HIV infection alone. Cervical carcinoma and its precursors share many risk factors with heterosexually acquired HIV infection, including substance abuse, non-white ethnicity, lower socioeconomic status, concordant STD infections, multiple sexual partners, and high risk heterosexual behavior. These epidemiologic risk factors for cervical carcinoma are likely additive, such that women with multiple risks may experience more rapid progression.[17] Coupled with a lack of any significant increase in the site-specific mortality from cervical cancer since the beginning of the AIDS epidemic,[18,19] our results lead one to suspect that HIV infection by itself may not necessarily result in more rapid progression of HPV-related disease in women.[20]

In conclusion, this report finds 3.3% of inner city women with abnormal cervical smears to be HIV seropositive at screening. Although high, this prevalence is lower than previous reports from similar urban areas where HIV infection is endemic. We found no evidence that cervical disease is more aggressive in newly diagnosed asymptomatic HIV-infected women than in HIV-seronegative women. From these data, we estimate that the risk of unknown HIV seropositivity in inner city women with abnormal Pap smears is highest between the ages of 20 and 49 years, in those who admit to exposure to HIV, and in those who are noted to have external condyloma on examination.

### REFERENCES

1. Laga M, Icenogle JP, Marsella R, et al.: Genital papillomavirus infection and cervical dysplasia: Opportunistic complications of HIV infection. Int J Cancer 50:45–48, 1992.
2. Caussy D, Goedert J, Palefsky J, et al.: Interaction of human immunodeficiency and papilloma viruses. Int J Cancer 46:214–219, 1992.
3. Schwartz LB, Carcangiu ML, Bradham L, Schwartz P: Rapidly progressive squamous cell carcinoma of the cervix coexisting with human immunodeficiency virus infection. Gynecol Oncol 41:255–258, 1991.
4. Maiman M, Fruchter RG, Serur E, Levine PA, Arrastia CD, Sedlis A: Recurrent cervical intraepithelial neoplasia in human immunodeficiency virus-seropositive women. Obstet Gynecol 82:1–5, 1993.
5. Maiman M, Fruchter R, Guy L, Cuthill S, Levine P, Serur E: Human immunodeficiency virus infection and invasive cervical cancer. Cancer 71:402–406, 1993.
6. Centers for Disease Control and Prevention: 1993 revised classification system for HIV infection and expanded surveillance case definition for AIDS among adolescents and adults. MMWR 41(RR-17):1–19, 1993.
7. Mandelblatt JS, Fahs M, Garibaldi K, Senie RT, Peterson HB: Association between HIV infection and cervical neoplasia: Implications for clinical care of women at risk for both conditions. AIDS 6:173–178, 1992.
8. ter Meulen J, Eberhardt H, Luande J, et al.: Human papillomavirus infection, HIV infection and cervical cancer in Tanzania, East Africa. Int J Cancer 51:515–521, 1992.
9. Maiman M, Fruchter R, Serur E, Boyce J: Prevalence of human immunodeficiency virus in a colposcopy clinic. JAMA 15:2214–2215, 1988.
10. Dottino PR, Sperling R, Kee R: Screening for human immunodeficiency virus and sexually transmitted diseases in an inner-city colposcopy clinic. Mount Sinai J Med 60:327–329, 1993.
11. Dawson-Saunders B, Trapp RG: Correlation and regression in: Basic and Clinical Biostatistics. Englewood Cliffs, NJ: Appleton & Lange, pp 168–170, 1990.
12. McCarthy K, Johnson M, Lawton F, Studd J: Acceptability of screening for HIV seroprevalence in women with cervical pathology. Lancet 340:1040–1041, 1992.
13. Spitzer M, Brennessel D, Seltzer VL, Silver L, Lox MS: Is human papillomavirus-related disease an independent risk factor for human immunodeficiency virus infection? Gynecol Oncol 49:243–246, 1993.
14. Lo B, Steinbrook R, Cooke M: Voluntary screening for HIV infection: Weighing the benefits and the harms. Ann Intern Med 110:727, 1989.
15. Fruchter RG, Maiman M, Sillman FH, Camilien L, Webber CA, Kim DS: Characteristics of cervical intraepithelial neoplasia in women infected with the human immunodeficiency virus. Am J Obstet Gynecol 171: 531–537, 194.
16. Smith JR, Kitchen VW, Botchery M, et al.: Is HIV infection associated with an increase in the prevalence of cervical neoplasia? Br J Obstet Gynaecol 100:149–153, 1993.
17. Rotkin ID: A comparison review of key epidemiologic studies in cervical cancer related to current searches for transmissible agents. Cancer Res 33:1353–1367, 1973.
18. Centers for Disease Control and Prevention: AIDS in women. MMWR 39:845–849, 1990.
19. McKenna MT, Buehler JW, Qualters JR, Chu SY: HIV and trends in cervical cancer death rates among young women. Am J Public Health 83:1792–1793, 1993.
20. Sperling R, Dottino PR: Review of "cervical neoplasia in women with HIV infection." Oncology 8:93–94, 1994.
